# Supplementary material for: Hydrothermal vents trigger massive phytoplankton blooms in the Southern Ocean
Source: Nat Commun. 2019 Jun 5;10:2451. doi: 10.1038/s41467-019-09973-6 (PMC6549147; doi:10.1038/s41467-019-09973-6)
Supplement: Supplementary file 1 — Supplementary Information [file 41467_2019_9973_MOESM1_ESM.docx]

Supplementary information:

**Hydrothermal vents trigger massive phytoplankton blooms in the Southern Ocean**

Ardyna et al.

**Supplementary Figure 1.** Intensity of zonal (top) and meridional (bottom) current velocity based on Argo deep displacement, from 2002 to 2016. Each dot corresponds to the location of one Argo float cycle (deep displacement between two profiles). Panels on the right show the frequency distribution of the duration of drifting period (top) and drifting depth (bottom).

**Supplementary Figure 2**. Box and whisker plots of the F490 factor calculated for each SO province (based on the temperature profiles following Pollard et al.^1^ and Swart et al.^2^; STZ: Subtropical zone, SAZ: Subantarctic zone, PFZ: Polar Frontal Zone, AAZ: Antarctic Zone, SACCZ: the zone south of the Antarctic Circumpolar Current). The median value is specified.

**Supplementary Note: Phytoplankton bloom distribution, type and biomass in the Southern Ocean (based on particle backscattering).**

Thanks to the BGC-Argo network, the characterization of phytoplankton biomass by FChla can be additionally completed with particle backscattering measurements (b_bp_), a proxy for suspended particles and Particulate Organic Carbon. On the basis of the high b_bp_ signatures of these hydrothermally-influenced blooms (Fig 2e and f), it is clear that enhanced chlorophyll *a* was not mainly driven by photoacclimation processes (i.e. increase of Chla per unit of phytoplankton carbon), but by a substantial increase in phytoplankton biomass. The magnitude of b_bp_ of these hydrothermally-influenced blooms is comparable to blooms in the vicinity of the islands (i.e. Crozet and Kerguelen islands) or for sea-ice edge blooms (Supplementary Figure 3). Therefore, the high b_bp_ signature that we observed in our study region provides additional evidence that these blooms are quantitively important. Note that, for comparison, these blooms are similar in magnitude to the productive blooms observed during the NABO8 experiment in the North Atlantic^3^.

**Supplementary Figure 3**. Map of the different bloom types (i.e., : HNLC; : island/plateau-influenced; : ocean ridge-influenced; : ice-influenced) sampled. The magnitude of the bloom (i.e., the maximum depth-integrated particle backscattering) is related to the size of the colored circles. The grey dots indicate the individual float profiles. The red, orange and grey zones are, respectively, shallow areas (>500 m), areas with downstream iron delivery, and areas characterized by a seasonal sea ice cover. Histograms of the frequency (b) and (c) boxplot according to the bloom type are displayed in relation to the bloom magnitude. In (c), the top and bottom limits of each box are the 25th and 75th percentiles, respectively. The lines extending above and below each box, i.e., whiskers, represent the full range of non-outlier observations for each variable beyond the quartile range. The results of the Kruskal–Wallis H test are shown in panel (c) and depict regions with statistically significant differences between the magnitudes of the bloom at the 95 % level (p < 0.05). *** denotes highly significant results (p < 0.0001).

**References**

1. Pollard, R. T., Lucas, M. I. & Read, J. F. Physical controls on biogeochemical zonation in the Southern Ocean. *Deep Sea Res. Pt. 2* **49**, 3289-3305, doi:10.1016/S0967-0645(02)00084-X (2002).
2. Swart, S., Speich, S., Ansorge, I. J. & Lutjeharms, J. R. E. An altimetry-based gravest empirical mode south of Africa: 1. Development and validation. *J. Geophys. Res.* **115**, 2156-2202, doi:10.1029/2009JC005299 (2010).
3. Briggs, N. et al. High-resolution observations of aggregate flux during a sub-polar North Atlantic spring bloom. *Deep Sea Res. Pt. 1* **58**, 1031-1039, [doi:10.1016/j.dsr.2011.07.007](doi:http://dx.doi.org/10.1016/j.dsr.2011.07.007) (2011).
